# Acute Pericarditis Due to SARS-COV-2 in a Kidney Transplant Recipient

**DOI:** 10.7759/cureus.16547

**Published:** 2021-07-21

**Authors:** Harpreet Gill, Paul Beinhoff, Morgan Lamberg, Tej Mehta

**Affiliations:** 1 Hospital Medicine, Medical College of Wisconsin, Wauwatosa, USA; 2 Internal Medicine, Medical College of Wisconsin, Wawautosa, USA; 3 Medicine, Medical College of Wisconsin, Wawautosa, USA; 4 Radiology, Medical College of Wisconsin, Wawautosa, USA

**Keywords:** sars-cov-2, covid 19, viral pericarditis, kidney transplant recipient, remdesivir

## Abstract

Severe acute respiratory syndrome coronavirus 2 (SARS-CoV-2) is highly contagious and typically presents with respiratory complications. Non-respiratory etiology has been increasingly identified in the literature, including cardiac manifestations. Here, we discuss an atypical case recently treated for SARS-CoV-2 presenting with acute pericarditis. Our patient’s medical history of autoimmune disease and renal transplant further complicated her care. There is currently no standard of therapy for SARS-CoV-2-related pericarditis. We would like to highlight increased awareness of this rare complication as well as successful treatment regimens for acute management of the disease.

## Introduction

Severe acute respiratory syndrome coronavirus 2 (SARS-COV-2) is the pathogen responsible for the COVID-19 pandemic which has caused over 179 million cases and 3.8 million deaths around the world as of June 2021 [1]. SARS-COV-2 is highly contagious and most severe in older patients and those with pre-existing comorbidities. The development and deployment of multiple vaccines have dramatically changed the landscape of the pandemic, but the management of long-term and atypical complications of the disease remains a challenge.

While SARS-COV-2 typically presents with respiratory symptoms like pneumonia, pathology in many other organ systems has been well documented. For example, cardiac complications of severe SARS-COV-2 have been increasingly identified in the literature. In a review of 11,500 hospitalized SARS-COV-2 patients, the myocardial injury was found in nearly a quarter of all cases [2]. Here, we present the case of acute pericarditis following aggressive SARS-COV-2 treatment in a kidney transplant recipient which was managed with colchicine and aspirin. 

## Case presentation

We present the case of a 71-year-old woman with a past medical history significant for systemic lupus erythematosus (SLE), focal segmental glomerulosclerosis, status post renal transplant 15 years prior, and type-2 diabetes mellitus. Her medications included tacrolimus and mycophenolate for pharmacologic immunosuppression following transplantation and sliding scale insulin.

She initially presented to the emergency department for care following four days of progressively worsening cough, shortness of breath with generalized weakness, chest tightness, fever, and chills. She was diagnosed with SARS-COV-2 pneumonia which was managed with a one-week course of 6 mg daily dexamethasone and remdesivir. Her symptoms steadily improved and she was discharged to home care with rivaroxaban prophylaxis following completion of treatment.

**Figure 1:**
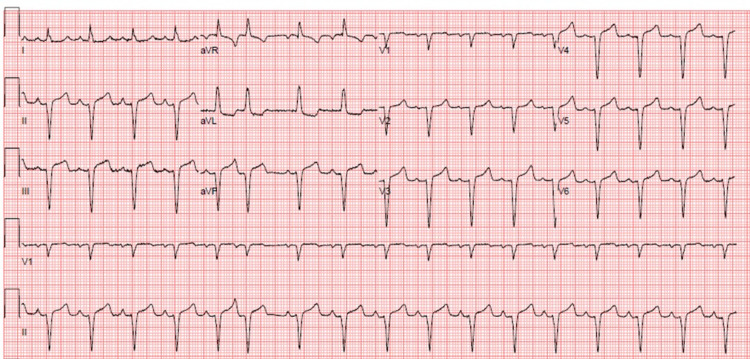
Electrocardiogram Following Readmission to Emergency Department Point of care EKG from her ED admission showing left axis deviation and premature atrial complexes in addition to pathognomonic features of pericarditis.

She represented to the emergency department the next day with shortness of breath, altered mental status, and chest pain which was exacerbated on inspiration. Her vitals on presentation were notable for a blood pressure of 172/81. Her physical examination was notable for an audible friction rub over her anterior chest wall and chest pain reproducibly improved by leaning forward. An electrocardiogram was obtained (Figure 1), which showed ST-segment elevations with PR interval depressions in leads II, III, aVF, and leads V3-V6. Additionally, her troponin was elevated at 283 ng/L which trended downward to 145 ng/L after two hours. Given her history of SLE, inflammatory markers were studied and showed elevations of C-​reactive protein (CRP) and erythrocyte sedimentation rate (ESR) at 1.6 mg/dL and 82 mm/hour, respectively. These findings suggested an infectious etiology, rather than an SLE exacerbation, as the reason for the inflammatory marker increase. Her other laboratory workup on presentation was unremarkable. Her anion gap was normal. A chest X-ray and transesophageal echocardiogram were also conducted due to suspicion for acute pericarditis but showed no signs of pleural effusion or pericardial effusion, respectively.

Given this constellation of findings, she was admitted to the hospital with presumptive pericarditis secondary to SARS-COV-2 infection. Management of pericarditis was achieved with 650 mg aspirin three times daily and 1.2 mg colchicine twice daily. Her cognition and chest pain markedly improved after the initiation of these therapies along with supportive treatment. Colchicine was reduced to 0.6 mg twice daily after one day. She was discharged two days after treatment in stable condition with 0.6 mg colchicine twice daily for three months and 650 mg aspirin three times daily to be tapered over two to four weeks following symptom resolution.

## Discussion

Members of the Coronaviridae family have been notable causes of viral outbreaks over recent decades, such as the causative agent of the SARS-COV-2 pandemic. SARS-COV-2 infection is frequently associated with respiratory symptoms, fever, loss of taste or smell, muscle pain, headache, and malaise. While many cases can lack symptoms altogether, severe cases can progress into an array of presentations which commonly include pneumonia, acute respiratory distress syndrome (ARDS), sepsis, and acute kidney injury [3].

There is a growing body of literature regarding cardiac complications from the virus. A recent prospective study conducted in Germany in non-selected participants found cardiac complications in 78% of recovered cases [4]. These complications were detected using cardiovascular MRI (CMR) and found that the most common complication was myocardial inflammation. A separate review of over 11,500 hospitalized patients found that acute myocardial injury was seen in nearly a quarter of all SARS-COV-2 positive patients [2].

Cardiac injury or carditis in SARS-COV-2 positive patients can be measured through serial Troponin 1 (cTnI) in concordance with inflammatory markers, CMR, and RT-PCR viral load of SARS-CoV2 RNA [5]. cTnI elevation in such patients has been associated with increased mortality, severity of disease, and intensive care requirements [6]. Pericarditis has been increasingly documented in the literature in SARS-CoV2 positive patients, with pleuritic chest pain as a primary complaint, as presented in the above case [7]. Cardiac involvement is not well correlated with pulmonary involvement and pericardial fluid detection of SARS-CoV2 RNA is most often negative, supporting the theory that pericardial involvement is primarily an inflammatory process [5]. Lastly, previous cardiac history and structural abnormalities are a potential risk factor for SARS-COV-2-related myopericarditis [5].

Injury has been identified to take place through a variety of mechanisms, including direct viral binding to the angiotensin-converting enzyme 2 (ACE-2) receptor, overactive cytokine production, and pathologic activation of the innate immune system [8]. The virus is able to directly bind ACE-2 receptors on cardiac tissue and antagonize the protective downstream signaling; ultimately leading to unopposed angiotensin II expression [2,9]. In the lungs, the loss of anti-inflammatory signaling leads to the destruction of alveolar tissue causing acute lung injury, hypoxemia, or ARDS which places cardiac tissue at risk for ischemia. Furthermore, the activation of macrophages and CD8+ cells leads to pathologic increases in cytokines. The effects of the cytokine storm include endothelial inflammation, microthrombi due to hypercoagulability, increased phagocytic infiltration, and possible heart failure [2,8].

Solid organ transplant recipients, such as the case presented above, are in a vulnerable and less studied population with regards to infectious diseases, such as SARS-COV-2. The immunocompromised status of transplant recipients adds uncertainty in prognosis and best practices. In their five-month review, Alfishawy et al. found 320 cases of SARS-COV-2 in transplant recipients to provide guidelines in treatment and immunosuppressive management [10]. As noted in their study, immunocompromised individuals are likely to present atypically and have impaired antiviral immunity justifying concern for higher expected mortality. Conversely, immunosuppression therapy could be a protective factor by decreasing the severity of the cytokine storm leading to mixed recommendations on whether to suspend therapy. Of the 220 kidney recipients, the mortality was approximately 20%, which is higher than the rate observed in the general population [10]. In addition to vulnerability from being immunosuppressed, this observed difference could be attributed to the higher age and number of comorbidities in transplant recipients when compared to the general population. With regards to the management of immunosuppression, multiple approaches including complete suspension of medications were attempted. The continuation of therapy in selected transplant patients was supported by the findings and the authors noted that discontinuing immunosuppression could result in acute rejection ultimately worsening the overall prognosis [10].

Current frontline treatment for SARS-COV-2 in severe cases includes concomitant remdesivir, an RNA-dependent-RNA-polymerase inhibitor, and dexamethasone. Evidence to support dexamethasone’s use comes from the RECOVERY trial in which 28-day mortality was improved; evidence supporting the use of remdesivir comes from a documented decreased time to recovery and lesser incidence of serious adverse events [11,12]. Treatment for pericarditis typically involves colchicine, an antimitotic, anti-inflammatory medication, with adjuvant aspirin administration. Evidence to support this regimen comes from documented decreased recurrence of acute pericarditis, symptom improvement, and clinical remission [13]. A multitude of international clinical trials also indicates that colchicine poses improved outcomes for SARS-COV2 pericarditis specifically [14]. A successful response to colchicine and concomitant aspirin therapy are also noted from recent case reports in addition to the case presented above [15]. Steroidal therapy is contraindicated in patients with pericarditis, due to concerns for an increased risk of recurrence [16].

## Conclusions

Acute pericarditis is a rare complication of SARS-COV-2. A high clinical suspicion should be noted in patients with recent SARS-CoV-2 positive assays in correlation with elevated troponins and EKG changes. Cardiac injury is hypothesized to occur secondary to immune response rather than direct toxicity. Treatment is currently case by case due to a lack of standardized therapy. Aggressive management including early hospitalization and adjuvant remdesivir, aspirin, and colchicine were successful for our patient.
